# The USP18 cysteine protease promotes HBV production independent of its protease activity

**DOI:** 10.1186/s12985-020-01304-2

**Published:** 2020-04-05

**Authors:** Yujia Li, Min Yao, Xiaoqiong Duan, Haiyan Ye, Shilin Li, Limin Chen, Chunhui Yang, Yongjun Chen

**Affiliations:** 1 Institute of Blood Transfusion, Chinese Academy of Medical Sciences and Peking Union Medical College, Chengdu, 610052 Sichuan China; 2 The University of Hong Kong Shenzhen Hospital, Shenzhen, 518053 China; 3 Toronto General Research Institute, University Health Network, University of Toronto, Toronto, Ontario M5G1L6 Canada

**Keywords:** HBV, Interferon, USP18, Type I IFN signaling pathway, Persistent infection

## Abstract

**Background:**

Hepatitis B virus (HBV) infection remains as one of the major public health problems in the world. Type I interferon (IFN) plays an essential role in antiviral defense by induced expression of a few hundred interferon stimulated genes (ISGs), including ubiquitin-specific protease 18 (USP18). The expression level of USP18 was elevated in the pretreatment liver tissues of chronic hepatitis B(CHB) patients who did not respond to IFN treatment. Thus, this study was designed to investigate the effects of USP18 on HBV replication/production.

**Methods:**

The levels of wild type USP18(WT-USP18) and USP18 catalytically inactive form C64S were up-regulated by plasmids transfection in HepAD38 cells, respectively. Real-time PCR and ELISA were used to quantify HBV replication. Type I IFN signaling pathway was monitored at three levels: p-STAT1 (western Blot), interferon stimulated response element (ISRE) activity (dual luciferase assay) and ISGs expression (real time PCR).

**Results:**

Our data demonstrated that overexpression of either WT-USP18 or USP18-C64S inactive mutant increased the intracellular viral pgRNA**,** total DNA, cccDNA, as well as HBV DNA levels in the culture supernatant, while silencing USP18 led to opposite effect on HBV production. In addition, upregulated WT-USP18 or USP18-C64S suppressed ISRE activity and the expression levels of p-STAT1 and ISGs.

**Conclusion:**

USP18 promoted HBV replication via inhibiting type I IFN signaling pathway, which was independent of its protease activity.

## Background

As one of the major public health problems, Hepatitis B virus (HBV) infected about 257 million people worldwide, leading to hepatic fibrosis, cirrhosis, and even hepatocellular carcinoma (HCC). Type I interferons (IFNs), the key effector of innate immunity, have long been used to treat different viral diseases such as HBV and Hepatitis C virus (HCV) infections. Although the functions of HBV proteins are increasingly understood, the role of host factors in modulating HBV infection remains elusive.

Increasing evidence puts IFN stimulated gene 15/ ubiquitin-specific protease 18 (ISG15/USP18) system, both of which are abundantly induced by type I IFN, at the center of regulatory processes of host innate immune response (reviewed in [1]). ISG15 plays its role in anti-viral defense and immunoregulation during pathogen evasion either through conjugating to host or pathogen proteins (ISGylation) [2] or function as a cytokine in free form [3]. As the major ISG15 de-conjugating enzyme, USP18 is an off-switch in ISGylation, striping ISG15 from its target proteins (known as deISGylation) and playing important role in viral infections through its ISG15 protease activity [4]. However, recent evidence suggested that USP18 might play its role independent of the protease activity: USP18 was able to bind to the subunit 2 of type I IFN receptor to inhibit IFN-induced Janus kinase/signal transducer and activator of transcription (Jak/STAT) signaling pathway [5].

Our previous data [6, 7] showed that chronic HCV patients, who had high pretreatment hepatic expression of a subset of interferon stimulated genes (ISGs), including ISG15 and USP18, did not respond to standard pegylated IFNα/Ribavirin (PegIFN/Rib) treatment. Similarly, our clinical data revealed that chronic hepatitis B (CHB) patients who had pre-treated higher hepatic USP18 expression responded poorly (or called “non-response, NR”) to IFN treatment [8]. Several groups also investigated the effects of USP18 suppression on HBV replication. Using in vivo HBV replication mouse model, Kim JH et al. [9] demonstrated that blocking ISGylation by UBE1L silencing exhibited very little effect on HBV replication, while USP18 knockout significantly suppressed HBV production. One more recent study [10] reported that silencing USP18 in HepG2.2.15 cells decreased the intracellular HBV pgRNA level and inhibited secretion of HBV DNA, HBsAg and HBeAg into the supernatant. However, very little study focused on the influence of USP18 overexpression on HBV replication/production. In the present study, we explored whether increased USP18 expression affected HBV replication in HepAD38 cells which, to some extent, resembled the persistent viral replication situation of the NR patients with high expression of USP18 in liver.

## Materials and methods

### Cell lines

HepG2 cells and Hela cells were cultured in Dulbecco’s Modified Eagle’s Medium (DMEM) (Gibco, USA) supplemented with 10% fetal bovine serum (FBS) (Gibco, USA), penicillin (100 IU/ml; Gibco, USA), and streptomycin (100 μg/ml; Gibco, USA) in 5% CO2 incubator at 37 °C. HepAD38 cells, derived from HepG2 cells by integrating full-length HBV genome in the cellular genome, support HBV expression under the control of a tetracycline-regulated (tet-off) promoter [11], were kindly provided by Professor Bo Qin and Dr. Zeng Tu (Chongqing Medical University, China). The cells were routinely cultured as mentioned above. Additionally, HepAD38 cells were treated with G418 for resistance screening.

### Plasmids construction

Human full-length USP18 gene fused to GFP at the N-terminus was cloned into pcDNA-DEST53 (Invitrogen, USA). Briefly, human USP18 ORF (in pENTER221 entry vector, Invitrogen, USA) was cloned into the destination vector (PcDNA-DEST53) by LR recombination (wide type USP18, WT-USP18). Western blot was used to confirm USP18 protein expression (anti-USP18 antibody). The USP18 mutant form C64S (USP18-C64S) was obtained by in vitro mutagenesis using the GeneTailor kit (Invitroge, USA). The primers used were: USP18 forward for C64S 5′-caacattggacagaccagctgccttaactccttga-3′ and USP18 reverse for C64S 5′-ggtctgtccaatgttgtgtaaaccaaccaggccat-3′. After methylation, pENTER221-USP18 was used as a template for PCR reactions using the above mutant primer pairs, and the resulting mutant form was screened on LB/Agar plates containing 100 μg/ml kanamycin. All constructs generated in this study were confirmed by DNA sequencing across joints.

### Confirmation of ISG15-cleavage activity of USP18 in vitro

ISG15/glutathione S-transferase (GST) expression plasmid was created by cloning the ISG15/GST fusion gene into pcDNA4/HisMax vector (Invitrogen, USA). Real-time PCRs was performed by employing faststart Universal SYBR Green Master Mix (Roche, USA) following the manufacturer’s protocols to determine expressions of ISG15 which was further confirmed by Western blot [12]. ISG15-cleavage activity of wild type USP18 (WT-USP18) and USP18-C64S were analyzed by co-transfection of ISG15/GST and WT-USP18 (or USP18-C64S) into Hela cells.

### Enyzme-linked immunosorbent assay (ELISA)

The levels of HBsAg and HBeAg in the culture supernatant of HepAD38 were detected by Human HBsAg and HBeAg ELISA Kits (Andy Gene, China) following the manufacturer’s instructions. The expression levels of IFNα and IFNβ in culture supernatant of HepG2 cells and HepAD38 cells were measured by Human IFNα/IFNβ ELISA Kits (Shanghai jijin Chemistry Technology, China),respectively.

### USP18 knockdown

USP18 small inhibitory RNA (siUSP18: 5′-CUGCAUAUCUUCUGGUUUATT-3′) and the negative control (NC) siRNA (NC: 5′-UUCUCCGAACGUGUCACGUTT-3′) were purchased from Sangon Biotech, China. HepAD38 cells were seeded at 3 × 10^5^/ml, 2 ml per well in 6-well plates. 24 h later, the cells were left untreated or transfected with 20 nM siUSP18 or 20 nM NC by using Lipofectamine® RNAiMAX (Invitrogen, USA) reagent according to the manufacturer’s instructions. 48 h post transfection, intracellular total protein was extracted to detect USP18 expression by western blot. Intracellular total RNA and DNA, as well as supernatant DNA, were collected respectively. Real-time PCR was performed to detect HBV pgRNA, USP18 mRNA, cccDNA and intracellular/supernatant HBV total DNA.

### RNA/DNA isolation and quantitative real-time polymerase chain reaction

Total RNA was extracted from HepAD38 cells using Trizol (Invitrogen, USA) and the cDNAs were synthesized by reverse transcription using ReverTraAce qPCR RT Master Mix (TOYOBO, Japan) following the manufacture’s protocol. Quantitative real-time PCR was performed using the SYBR Green Real-time PCR Master Mix (TOYOBO, Japan). Total intracellular DNA in cells were acquired by TIANamp Genomic DNA Kit (TIANGEN, China) while supernatant viral DNA were extracted by QIAamp DNA Blood Mini Kit (Qiagen, German). The forward and reverse primers specific for the genes are shown in Table 1. HBV DNA, pgRNA, and some ISGs were quantified by real-time PCR according to the instructions.

**Table 1:** Real-Time PCR Primes

Gene Name	Nucleotide sequence
GAPDH	Forward 5′-GCCTCCTGCACCACCAACTG-3′ Reverse 5′-ACGCCTGCTTCACCACCTTC-3′
USP18	Forward 5′-CAGACCCTGACAATCCACCT-3′ Reverse 5′-AGCTCATACTGCCCTCCAGA-3′
HBV total DNA	Forward 5′-CGTTTTTGCCTTCTGACTTCTTTC-3′ Reverse 5′-ATAGGATAGGGGCATTTGGTGGTC-3′
HBV cccDNA	Forward 5′-TTCTCATCTGCCGGACCG-3′ Reverse 5′-CACAGCTTGGAGGCTTGAAC-3’
HBV pgRNA	Forward 5’-CTCAATCTCGGGAATCTCAATGT-3′ Reverse 5′-TGGATAAAACCTAGCAGGCATAAT-3’
MxA	Forward 5’-GTGCATTGCAGAAGGTCAGA-3′ Reverse 5′-CTGGTGATAGGCCATCAGGT-3’
OAS2	Forward 5’-TCAGCGAGGCCAGTAATCTT-3′ Reverse 5′-GCAGGACATTCCAAGATGGT-3’

*USP18* Ubiquitin specific protease 18, *HBV cccDNA* hepatitis B virus covalently closed circular DNA, *pgRNA* hepatitis B virus pregenomic RNA, *MxA* myxovirus resistance protein A, *OAS2* 2’,5′-oligoadenylate synthetase 2

### Western blot analysis

Western blot was performed to detect the semi-quantification of ISG15、USP18 and HBV protein levels. The primary antibodies used were as follows, rabbit anti-ISG15 (CST, USA), mouse anti-GAPDH (Zheng De, China), mouse anti-β-actin (CST, USA), mouse anti-HBcAg (Boster Biological Technology, China), rabbit anti-p-STAT1 (Tyr701) (CST, USA) and rabbit anti-STAT1 (CST, USA). For USP18, two kinds of primary antibodies were used: USP18 Polyclonal Antibody (Invitrogen, USA) (one band) and rabbit anti-USP18 (CST, USA) (two bands). Secondary antibodies were HRP-labeled goat anti-mouse (Biosharp, China) or anti-rabbit IgG (Beyotime, China). The protein bands were visualized using an ECL chemiluminescent detection kit (Millipore, USA) by ChemiDocTM Imaging Systerm (BIO-RAD, USA). The relative intensities of protein bands were analyzed with ImageJ2 × 2.1.4.7 software.

### Dual-luciferase report gene system

HepAD38 cells were seeded at a density of 3.0 × 10^5^ per well in 24-well plates. Twenty-four hours later, 0.5 μg ISRE (interferon stimulated response element)-luc reporter plasmid and 2 ng PRL-TK reporter plasmid were co-transfected with 1 μg pcDNA3.1–3*tag plasmid (MOCK) or 1 μg USP18 plasmid. Twelve hours after transfection, the culture medium was removed and replenished with fresh medium. Twenty-four hours post transfection, cells were treated with IFNα (0 IU/ml, 100 IU/ml and 1000 IU/ml) for additional 24 h. Then, cells were lysed with passive lysis buffer and the relative luciferase activity was detected by Dual-Luciferase Reporter(DLR) Assay kit (Promega, USA) according to the manufacturer’s protocol.

### Statistical analysis

All experiments in this study were performed at least three independent times. Statistical differences were compared by Student’s t-test through GraphPad Prism software*. p* values≤0.05 were considered statistically significant.

## Results

### Confirmation of USP18 expression and its catalytic activity

**Fig. 1:**
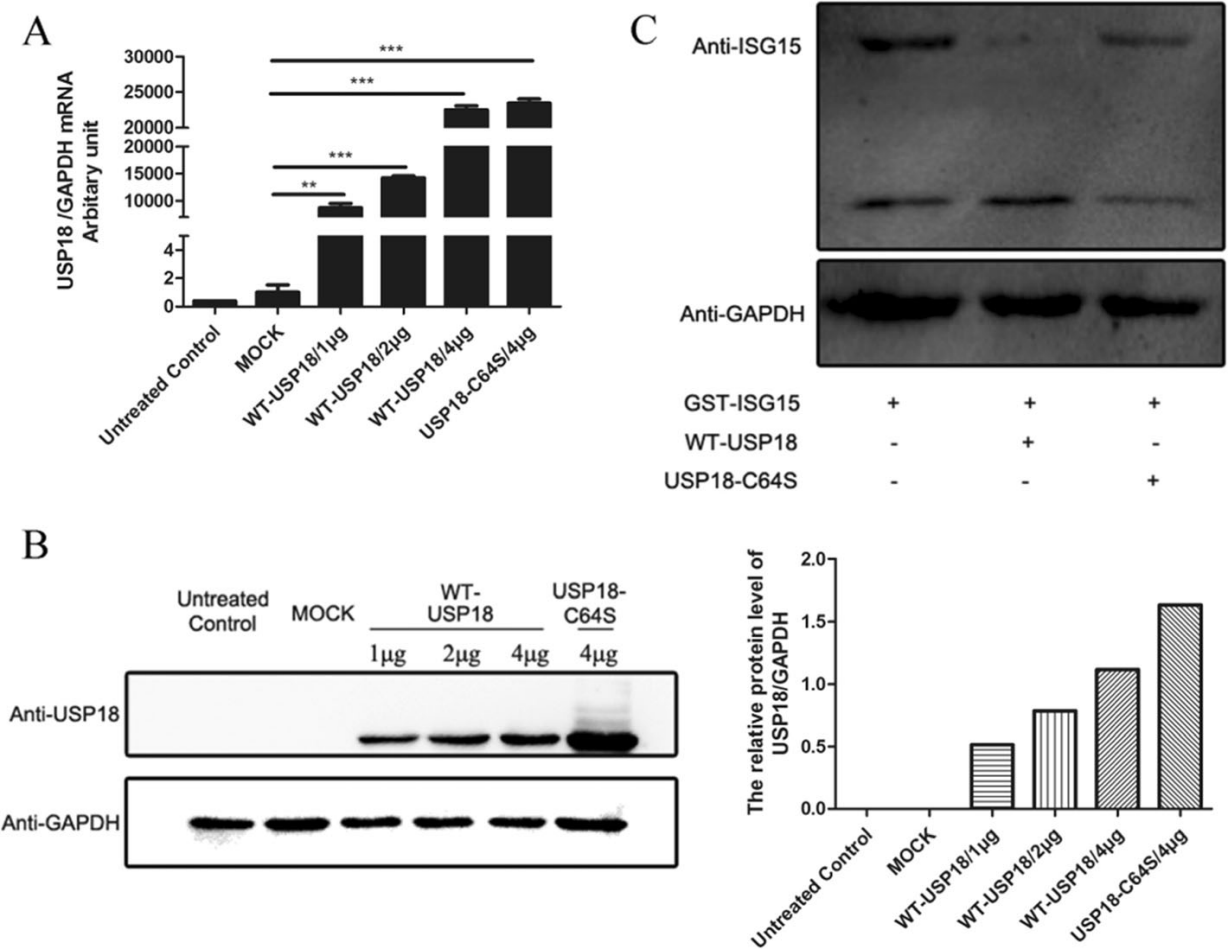
Over-expression of USP18 and its catalytic activity. HepAD38 cells were transfected with WT-USP18 plasmid, USP18-C64S plasmid or empty vector (MOCK) or left untreated. **a**: Twenty-four hours after transfection, USP18 mRNA was determined by real-time PCR (normalized by GAPDH). **b**: Forty-eight hours after transfection, USP18 protein expressions were analyzed by western blot (left). The relative expression levels of USP18 (normalized by GAPDH) were calculated by densitometry analysis (right). **c**: Cleavage of ISG15-GST fusion in vitro. USP18, ISG15/GST and WT-USP18 (or USP18-C64S) were co-transfected into Hela cells. Total intracellular protein was collected to perform Western blot. WT-USP18, wide type USP18; MOCK, empty plasmid. Results are presented as means ± SD (*n* ≥ 3). ** p ≤ 0.01; *** p ≤ *0.001*

In order to explore the effect of USP18 on HBV infection, we first confirmed whether USP18 and USP18-C64S-ecoding plasmids were successfully constructed. Figure 1a showed that transfection of WT-USP18 or USP18-C64S plasmid led to a pronounced increase of USP18 mRNA expression in a dose-dependent manner, which was further confirmed by western blot (Fig. 1b). The transfection efficiency was shown by the GFP expression in the cells (Supplement Fig. 1). Since it has been reported [13] that full length USP18 has a conserved catalytic-activity-related site cysteine at Cys64 in its Cys-box, we obtained the mutant form of USP18 by conversing the cyserine into serine. And then the Hela cells, in which ISGylation could not be induced because of lacking E1 activating enzyme Ube1L [14], were co-transfected with the pcDNA4/HisMax-ISG15/GST and WT-USP18 or USP18-C64S plasmids. Western blot showed two bands of ISG15: the upper GST-ISG15 band and the lower ISG15 band, which indicated the expression of WT-USP18 led to release of the ISG15 protein from its conjugated GST-ISG15, while USP18-C64S did not (Fig. 1c)**.**

### USP18 regulated HBV production independent of its protease activity

**Fig. 2:**
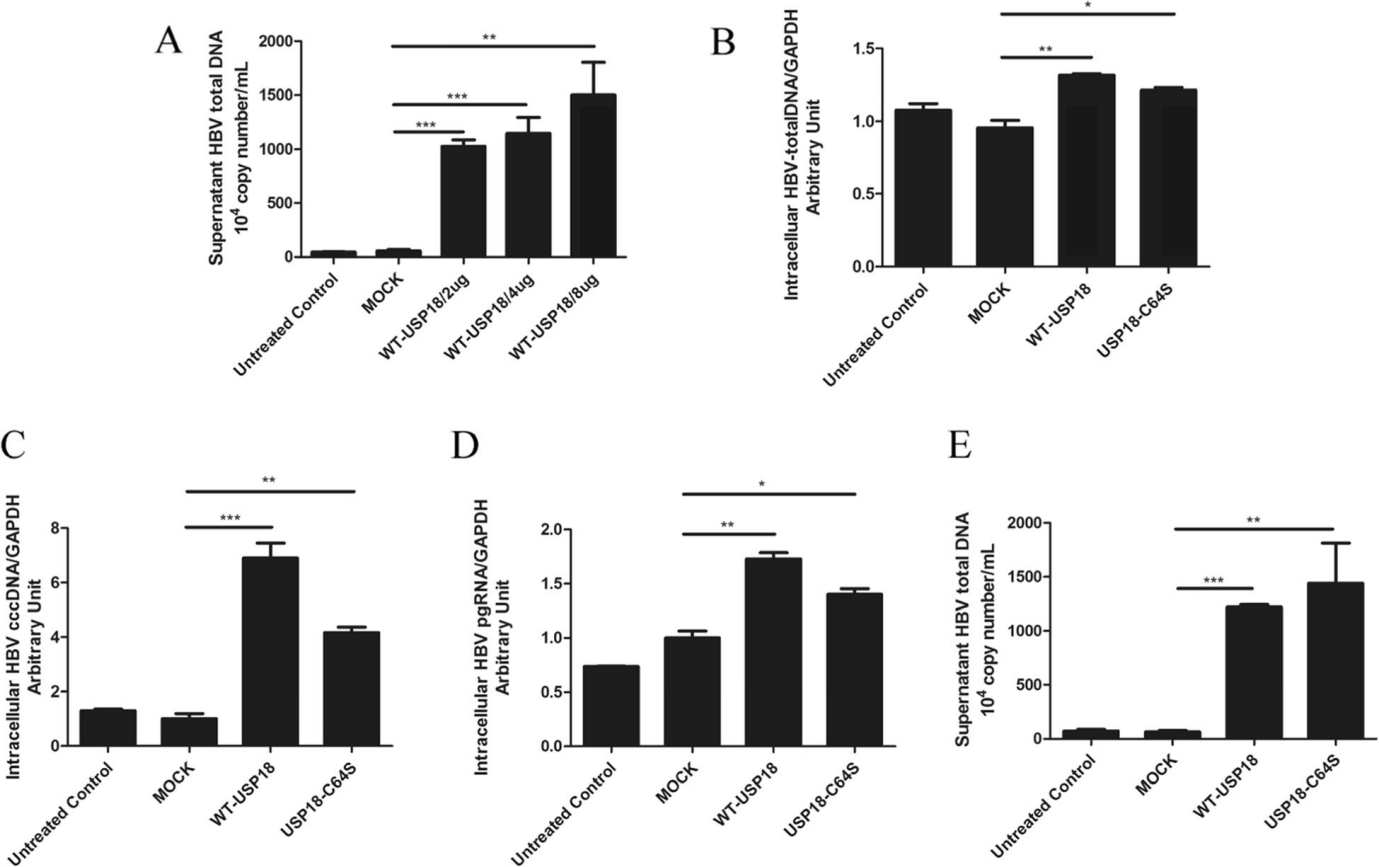
Over-expression of USP18 in HepAD38 cells promoted HBV production independent of its protease activity. HepAD38 cells were transfected with USP18 plasmids as indicated. Real-time PCR was performed to quantify HBV production. Total supernatant HBV DNA (**a**), total intracellular HBV DNA (**b**), HBV cccDNA (**c**) and HBV pgRNA (**d**) 48 h post transfection with 4 μg WT-USP18 plasmid. Supernatant HBV total DNA 48 h post transfection with 4 μg WT-USP18 or 4 μg USP18-C64S plasmid, respectively (**e**). WT-USP18, wide type USP18; MOCK, empty plasmid. Results are presented as means ± SD (n ≥ 3). * p ≤ 0.05; ** p ≤ 0.01; ***p ≤ *0.001*

**Fig. 3:**
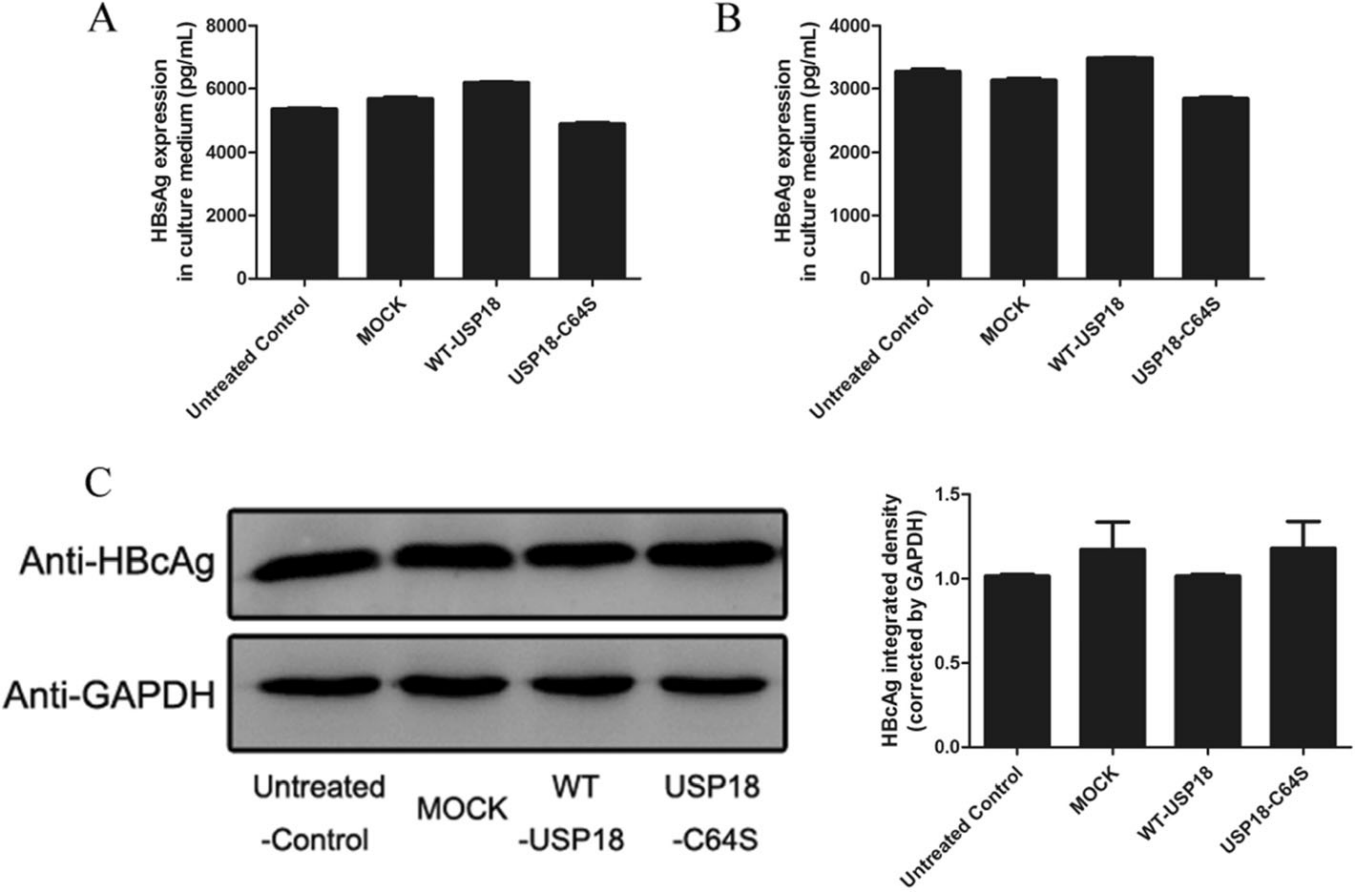
Over-expression of USP18 in HepAD38 cells did not affect expression of HBV proteins. HepAD38 cells were transfected with the 4 μg WT-USP18, 4 μg USP18-C64S or 4 μg MOCK, respectively. Forty-eight hours later, culture medium was collected to quantify HBsAg (**a**) and HBeAg (**b**) expression level by ELISA assay. Intracellular HBcAgwas detected by western blot (**c, left**) and (**c, right**) analyzed by densitometry analysis. WT-USP18, wide type USP18; MOCK, empty plasmid. Results are presented as means ± SD (n ≥ 3)

**Fig. 4:**
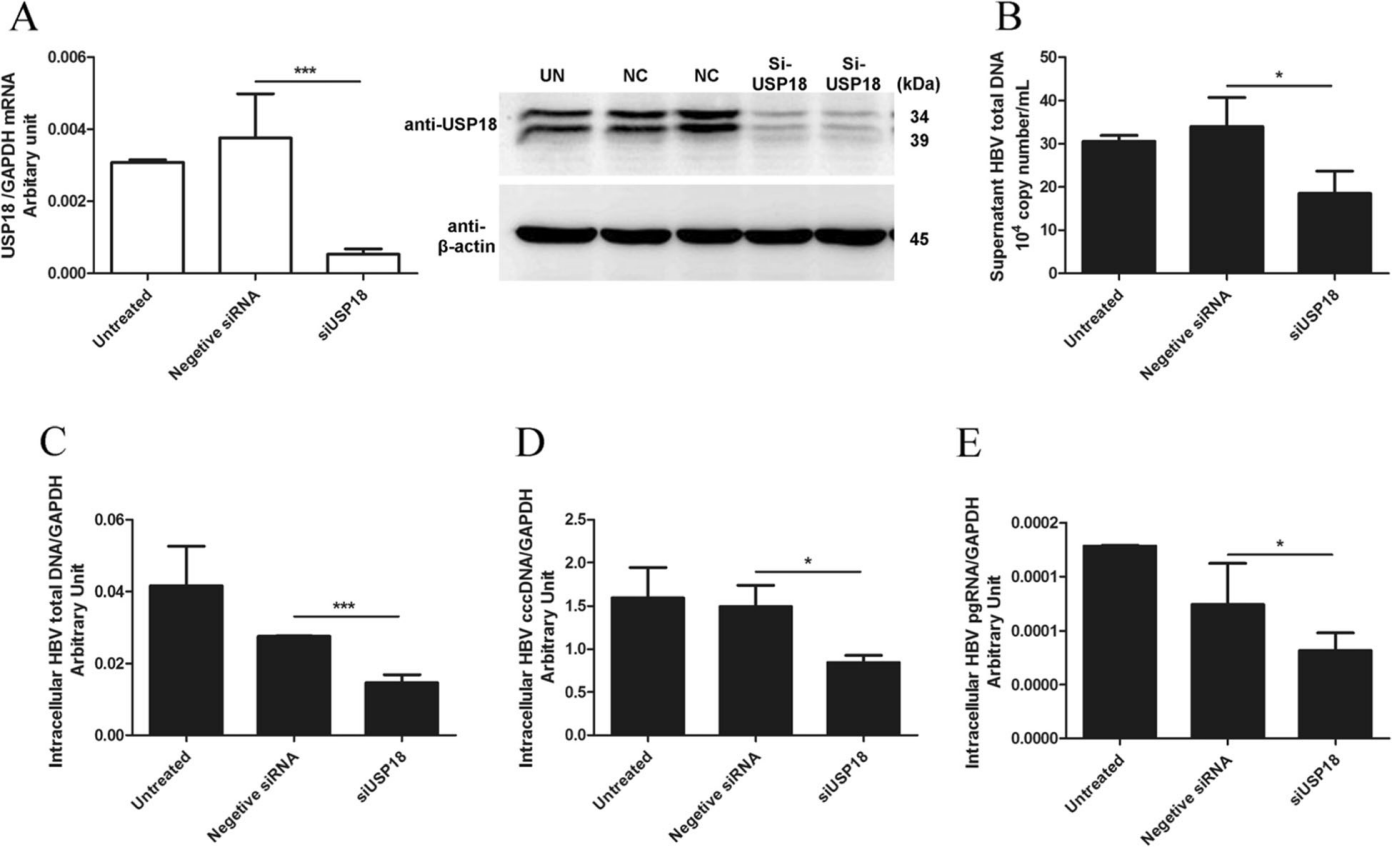
Silencing USP18 in HepAD38 suppressed HBV production. HepAD38 cells were seeded at 3 × 10^5^/ml, 2 ml per well in 6-well plates. 24 h later, the cells were left untreated or transfected with 20 nM siUSP18 or 20 nM NC. 48 h post transfection, knockdown efficiency was evaluated by detecting USP18 mRNA expression (**a, left**), which was further confirmed by western blot (**a, right**). Intracellular total RNA and DNA, as well as supernatant DNA, were collected respectively. Real-time PCR was performed to detect supernatant HBV total DNA (**b**), intracellular HBV total DNA (**c**), HBV cccDNA (**d**) and pgRNA (**e**). siUSP18, USP18 small inhibitory RNA; NC, the negative control siRNA. Results are presented as means ± SD (n ≥ 3). *p ≤ 0.05; **p ≤ 0.01; ***p ≤ *0.001*

To evaluate the effect of USP18 on HBV replication, we analyzed the expression levels of supernatant HBV DNA, intracellular HBV pgRNA, total HBV DNA and cccDNA, following USP18 over-expression in HepAD38 cells. The results showed that total supernatant HBV DNA was dramatically increased in parallel with USP18 up-regulation in a dose-dependent manner (Fig. 2a). And the expression of intracellular HBV DNA (Fig. 2b), cccDNA (Fig. 2c), as well as the pgRNA (Fig. 2d) were also elevated with ectopic USP18 expression. We then asked whether the catalytic ability of USP18 was responsible for enhanced HBV replication. As expected, either WT-USP18 or USP18-C64S catalytically inactive mutant could stimulate HBV production (Fig. 2e), indicating that USP18 stimulated HBV production independent of its proteolytic capacity. More interestingly, USP18 showed very limited effect on HBsAg (Fig. 3a), HBeAg (Fig. 3b) in the supernatant and Intracellular HBcAg (Fig. 3c). We also evaluated the effect of USP18 silencing on HBV production. USP18 mRNA expression was significantly suppressed by RNA interference (Fig. 4a, left), which was further confirmed by western blot (Fig. 4a, right). And as expected from the experimental design, the supernatant (Fig. 4b) and Intracellular (Fig. 4d) total HBV DNA, cccDNA (Fig. 4e), as well as pgRNA (Fig. 4f) levels were decreased significantly in parallel with USP18 silencing, while the HBsAg, HBeAg and intracellular HBcAg remained unaffected (Supplement Fig. 2).

### Over expression of USP18 inhibited IFN-induced Jak/STAT signaling pathway

**Fig. 5:**
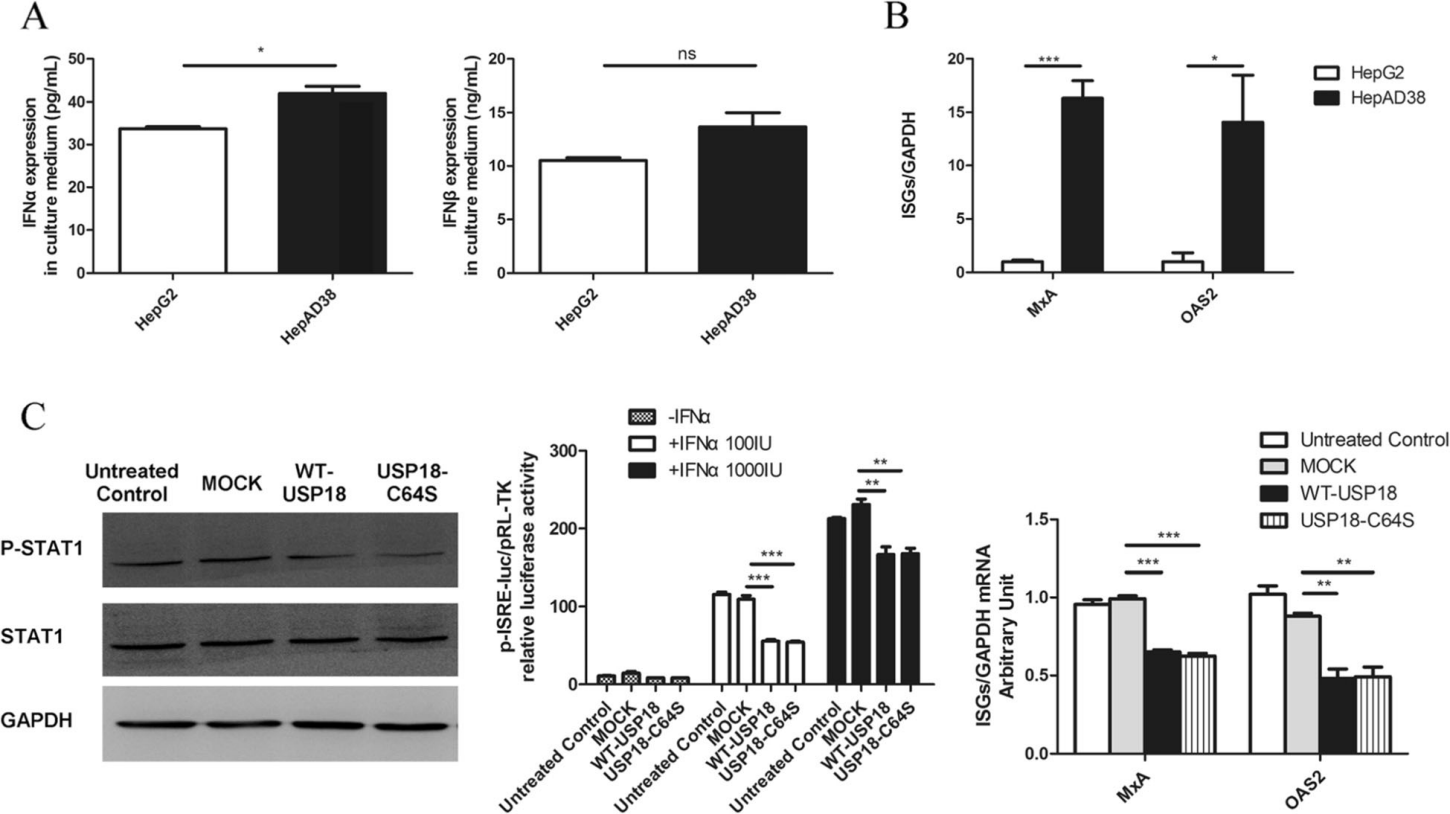
USP18 upregulation inhibited IFN-induced Jak/STAT signaling pathway. HepAD38 cells and HepG2 cells were seeded in 6-well plate without any treatment, respectively. Forty-eight hours later, supernatant IFNα (**a, left**) and IFNβ (**a, right**) and intracellular mRNA expression of ISGs (**b**) were analyzed by ELISA assay and real-time PCR, respectively. To investigate the effects of USP18 on STAT phosphorylation, HepAD38 cells were transfected with WT-USP18, USP18-C64S or MOCK for 48 h and treated with 500 IU/ml IFNα for 30 min before harvested. Western blot was used to analyze the expression of STAT1 and p-STAT1 (**c, left**). The interferon stimulated response element (ISRE) activity was quantified by dual luciferase reporter gene assay. Briefly, HepAD38 cells were co-transfected with WT-USP18, USP18-C64S or MOCK and ISRE-luc reporter plasmid /pRL-TK reporter plasmid for 24 h, and then left untreated or treated with 100 IU/ml or 1000 IU/ml IFNα for 24 h before the cells were lysed (**c, middle**). HepAD38 cells were transfected with WT-USP18, USP18-C64S or MOCK for 48 h and treated with 500 IU/ml IFNα for additional 24 h. Expression of ISGs mRNA including MxA and OAS2 were detected by real-time PCR (**c, right**). WT-USP18, wide type USP18; MOCK, empty plasmid. Results are presented as means ± SD (n ≥ 3). *p ≤ 0.05; **p ≤ 0.01; ***p ≤ *0.001*

Due to the fact that USP18 is a negative modulator of type I IFN signaling pathway, we hypothesize that endogenous type I IFN has been induced by HBV embraced in HepAD38 cells to defense viral replication and over-expression of USP18 may suppress the activation of Jak/STAT signaling pathway, consequently facilitating HBV production. In order to prove this hypothesis, we firstly measured the expression of type I IFN in HepAD38 and HepG2 cell culture medium by ELISA. As shown in Fig. 5a, although no difference was found in IFNβ expressions between these two cell lines, statistically significant potentiation of IFNα expression was found in HepAD38 cells compared with that in HepG2 cells. Real-time PCR further revealed that the expression of typical ISGs (MxA and OAS2) was increased in HepAD38 cells compared to HepG2 cells (Fig. 5b), indicating the activated type I IFN signaling pathway in HepAD38 cells. In consistent with this, when the tetracycline-responsive replication of HBV was inhibited by tetracycline(1 mg/ml) treatment, the endogenous IFNα and ISG mRNA including MxA and OAS2 expression were also depressed (Supplemental Fig. 3). We then asked whether USP18 overexpression could affect Jak/STAT signaling pathway in HepAD38 cells. Western blot, dual luciferase assay and real-time PCR were employed to evaluate the protein level of phosphorylated STAT1 (p-STAT1), ISRE activity and mRNA expression of typical ISGs, respectively. The results suggested that levels of p-STAT1 (Fig. 5c, left), ISRE activity (Fig. 5c, middle) and ISGs (MxA, OAS2) (Fig. 5c, right) were all inhibited by up-regulation of either WT-USP18 or USP18-C64S, indicating the impaired type I IFN signaling.

## Discussion

Previous work from several laboratories examined the role of USP18 in innate defense to HBV infection. USP18 has been proved to be associated with HBV infection in mouse model. Either USP18 knockout mice or USP18 knockdown mice generated by specific shRNA experienced restricted HBV replication, while the UbE1L−/− mouse which had suppressed ISG15 modification did not [9]. More recent evidence [10] suggested that silencing USP18 alone was enough to inhibit HBV expression in human HepG2.2.15 cells. In accordance with these studies, our study observed that over-expression of USP18 could promote HBV production in a catalysis-unconcerned manner, which, to some extent, provided an explanation to the association between high pre-treatment hepatic ISGs levels and persistent HBV infection [15]. Similarly, USP18−/− mice showed enhanced resistance to lymphocytic choriomeningitis virus (LCMV) or vesicular stomatitis virus (VSV) infection and reduced viral replication [16, 17]. Nevertheless, over-expression of USP18 significantly restrict viral replication during porcine respiratory and reproductive syndrome virus (PRRSV) infection which might be attributed to early activation of NF-κB [18, 19]. These diverse effects on virus infections implicate that USP18 functions via virus-specific and/or species-specific fashions which may be mediated by the interaction of USP18 and certain viral proteins.

Although responses vary between individuals [20, 21], type I IFN is still one of the most important choices in HBV therapy. Using different cell models, several groups [5, 22] reported that USP18 could interfere with the binding of IFNα to its receptor, resulting in cellular desensitization to further IFNα stimulation. In human cells, USP18 also have a similar role [22, 23]. Our data indicated that existence of HBV in HepAD38 cells stimulated the expression of endogenous type I IFN and subsequent signal pathway compared with that in HBV-free HepG2 cells. We speculated that this activated innate immune state, to some extent, can suppress HBV replication at a relatively low level. And over-expression of USP18 inhibited type I IFN signal pathway and broken the balance, resulting in elevated HBV replication. Furthermore, it is very interesting that overexpression of USP18 or USP18 C64S showed very little influence on intracellular HBcAg, supernatant HBsAg and HBeAg, while they promoted HBV DNA level significantly. At least two reasons might be involved: 1) The life cycle of HBV in HepAD38 cells (in vitro model) is not exactly the same as that in in vivo model or patients. With different treatment, the change of HBV nucleic acid level is not always in parallel with that of HBV protein expression levels in HepAD38 cells. It has been reported that HBV DNA in the culture medium was increased by doxycycline treatment [24] or tetherin [25] overexpression while the supernatant HBsAg level was not affected. And Kinoshita W et al. [26] found that host factor PRPF31 knockdown suppressed cccDNA production but showed little effect on HBcAg expression. 2) USP18 may affect HBV replication through different ways.

In contrast to HCV, acute HBV infection seldom induced type I or type III IFN production in chimpanzees [27]. Clinical data [28] also showed that patients with acute HBV infection did not experience significant IFN expression. This phenomenon may be caused by mild replication of HBV at early stage of infection [29]. However, it may be a little bit different in the chronic infection. First of all, JAK-STAT Signaling Pathway has been demonstrated to be involved in regulating HBV replication in HepG2.2.15 cells [10, 26] and HepAD38 cells [24, 25] with persistent HBV virion expression. Secondly, expression of IFN and upregulation of ISGs have been observed in persistent HBV infection. Dongni Gao et al. [30] found that after transfected with pHBV (containing the full-length genome of the HBV genotype C), the IFNα protein levels in Huh7 cells culture supernatants was up to 100 pg/Ml. Our previous study also showed that the pre-treatment expression of ISGs (ISG15, MxA, etc) was significantly up-regulated in hepatocytes of some patients with chronic hepatitis B [8, 15]. Most recently, Li L et al. [10] also reported that the expression level of USP18 in HepG2.2.15 cells was higher than that in HepG2 cells. Although more evidence is required to elucidate the potential mechanism, we could speculate that there might be a balance between the baseline activation of IFN signaling pathway and HBV replication. And once the balance is disturbed, the viral replication is promoted. Moreover, HBV DNA in the liver and blood could be cleared before onset of adaptive immune response [29], indicating that innate molecules, in addition to IFN, may contribute to controlling HBV infection. In fact, with exposure to HBV, macrophages could be activated characterizing by upregulated expression of inflammatory cytokines [31]. For example, interleukin 6 (IL-6) was reported to suppress early HBV gene expression by down-regulating the level of two transcription factors, hepatocyte nuclear factor (HNF) 1α and HNF 4α [32]. Thus, further study will be needed to investigate the effects of USP18 on these newly identified HBV-associated innate molecules.

It is wildly accepted that USP18 could conduct functions by controlling ISGylation dependent of its protease activity. Owing to the fact that over 300 cellular proteins [33, 34] and increasing number of pathogen’s proteins [35–38] have been identified as ISG15 targets, it is conceivable that USP18 is a key modulator involved in diverse cellular pathways and biological processes. On the other hand, however, there are multiple lines of evidence for protease-independent activities of USP18.Our findings revealed that either wide type USP18 or USP18 C64S could promote HBV production in HepAD38 cells. Klaus-Peter Knobeloch et al. [39] compared USP18−/− mice with ISG15−/− and USP18−/− double-deficient mice and found that ISG15 deletion did not rescue the severe consequences, such as high mortality, neurological symptoms and hydrocephalus, resulting from USP18 deficiency. Their findings indicated that although elevated ISGylation was induced by USP18 knockout, severe phenotype of USP18−/− mice might be caused by non-ISG15 mediating mechanism rather than lack of deISGylation. Harish Potu’s group [40] showed that both USP18 and its catalytically inactive form had similar anti-apoptotic effects to limit the apoptosis triggered by IFNα or bortezomib.

## Conclusions

Our results presented here showed that USP18 stimulated HBV production by inhibiting type I IFN signaling pathway independent of its protease activity. Although the precise mechanism and the involvement of host or viral molecules remain to be clarified, it raised the possibility of identifying USP18 as a potential therapeutic target in setting up a novel strategy of clinical protocols, especially for the treatment of HBV-infected patients.

## Supplementary information

**Additional file 1: Supplemental Figure 1. Transfection efficiency in HepAD38 cells.** HepAD38 cells were seeded at 3 × 10^5^/ml, 2 ml per well in 6-well plates in antibiotic-free medium for 24 h before 1μg (**A**), 2μg (**B**), 4μg (**C**) GFP plasmid DNA or 4μg empty vector (**D**) was transfected into each well. Fluorescent microscopy images were taken 48 h post transfection. **Supplemental Figure 2. USP18 knockdown in HepAD38 cells did not affect expression of HBV proteins.** HepAD38 cells were transfected with the 20 nM siUSP18, 20 nM negative siRNA or left untreated, respectively. Forty-eight hours later, culture medium was collected to quantify HBsAg **(A)** and HBeAg (**B**) expression level by ELISA assay. Intracellular HBcAg was detected by western blot (**C**). Results are presented as means ± SD(*n* ≥ 3). **Supplemental Figure 3. IFN and ISG expression in HepAD38.** HepAD38 cells were grown in the medium with or without tetracycline (1 mg/ml) until confluent for 6 days. Intracellular RNA was then extracted. Expression of IFNα (**A**), IFNβ (**B**) and ISGs mRNA including MxA (**C**) and OAS2 (**D**) were detected by real-time PCR. Results are presented as means ± SD (n ≥ 3). *p ≤ 0.05; **p ≤ 0.01; ***p ≤ 0.001. **Supplemental Figure 4. Detection of mycoplasma in HepAD38 cells.** HepAD38 cells were grown in the medium with or without tetracycline (1 mg/ml) until confluent. The supernatant was collected for detecting mycoplasma. NC, negative control; PC, positive control; Tet+, the complete HepAD38 medium with 1 mg/ml tetracycline; Tet-, the complete HepAD38 medium without tetracycline.

## Data Availability

All data generated or analysed during this study are included in this published article.

## Abbreviations

CHB: Chronic hepatitis B
GST: Glutathione S-transferase
HBV: Hepatitis B virus
HCC: Hepatocellular carcinoma
HCV: Hepatitis C virus
HNF: Hepatocyte nuclear factor
IFN: Interferon
IL-6: Interleukin 6
ISG15: Interferon stimulated gene 15
Jak/STAT: Janus kinase/signal transducer and activator of transcription
LCMV: Lymphocytic choriomeningitis virus
MxA: Myxovirus resistance-1
OAS2: 2′,5′-oligo adenylate synthetase 2
PegIFN/Rib: Pegylated IFNα/ Ribavirin
PRRSV: Porcine respiratory and reproductive syndrome virus (PRRSV)
USP18: Ubiquitin-specific protease 18
VSV: Vesicular stomatitis virus
WT-USP18: Wide type USP18
