# Supplementary material for: The USP18 cysteine protease promotes HBV production independent of its protease activity
Source: Virol J. 2020 Apr 5;17:47. doi: 10.1186/s12985-020-01304-2 (PMC7133002; doi:10.1186/s12985-020-01304-2)
Supplement: Supplementary file 1 — Additional file 1: Supplemental Figure 1. Transfection efficiency in HepAD38 cells. HepAD38 cells were seeded at 3 × 10^5^/ml, 2 ml per well in 6-well plates in antibiotic-free medium for 24 h before 1μg (A), 2μg (B), 4μg (C) GFP plasmid DNA or 4μg empty vector (D) was transfected into each well. Fluorescent microscopy images were taken 48 h post transfection. Supplemental Figure 2. USP18 knockdown in HepAD38 cells did not affect expression of HBV proteins. HepAD38 cells were transfected with the 20 nM siUSP18, 20 nM negative siRNA or left untreated, respectively. Forty-eight hours later, culture medium was collected to quantify HBsAg (A) and HBeAg (B) expression level by ELISA assay. Intracellular HBcAg was detected by western blot (C). Results are presented as means ± SD(n ≥ 3). Supplemental Figure 3. IFN and ISG expression in HepAD38. HepAD38 cells were grown in the medium with or without tetracycline (1 mg/ml) until confluent for 6 days. Intracellular RNA was then extracted. Expression of IFNα (A), IFNβ (B) and ISGs mRNA including MxA (C) and OAS2 (D) were detected by real-time PCR. Results are presented as means ± SD (n ≥ 3). *p ≤ 0.05; **p ≤ 0.01; ***p ≤ 0.001. Supplemental Figure 4. Detection of mycoplasma in HepAD38 cells. HepAD38 cells were grown in the medium with or without tetracycline (1 mg/ml) until confluent. The supernatant was collected for detecting mycoplasma. NC, negative control; PC, positive control; Tet+, the complete HepAD38 medium with 1 mg/ml tetracycline; Tet-, the complete HepAD38 medium without tetracycline. [file 12985_2020_1304_MOESM1_ESM.doc]

**Supplemental Figure 1**

**
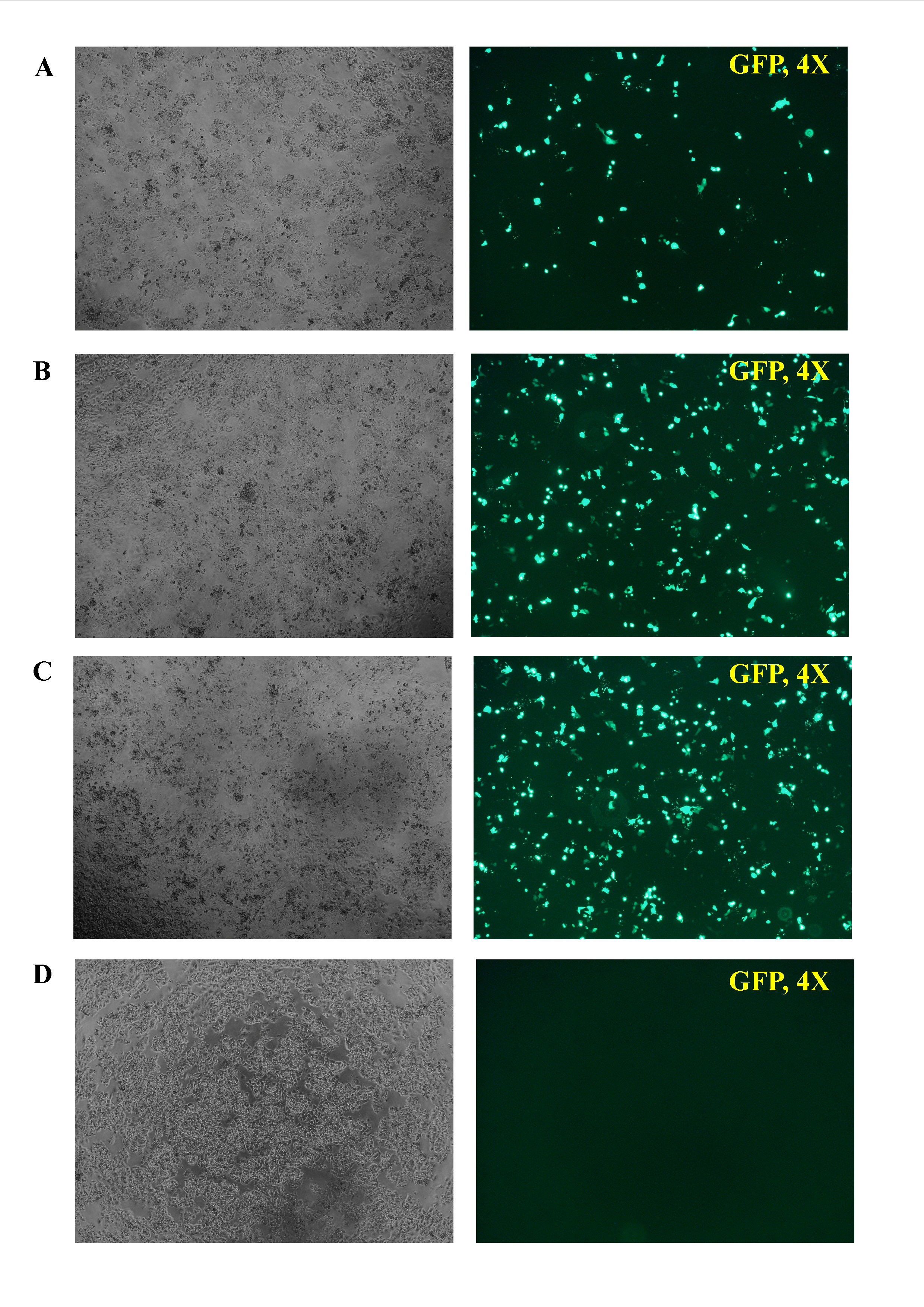
**

**Supplemental Figure 2**

**
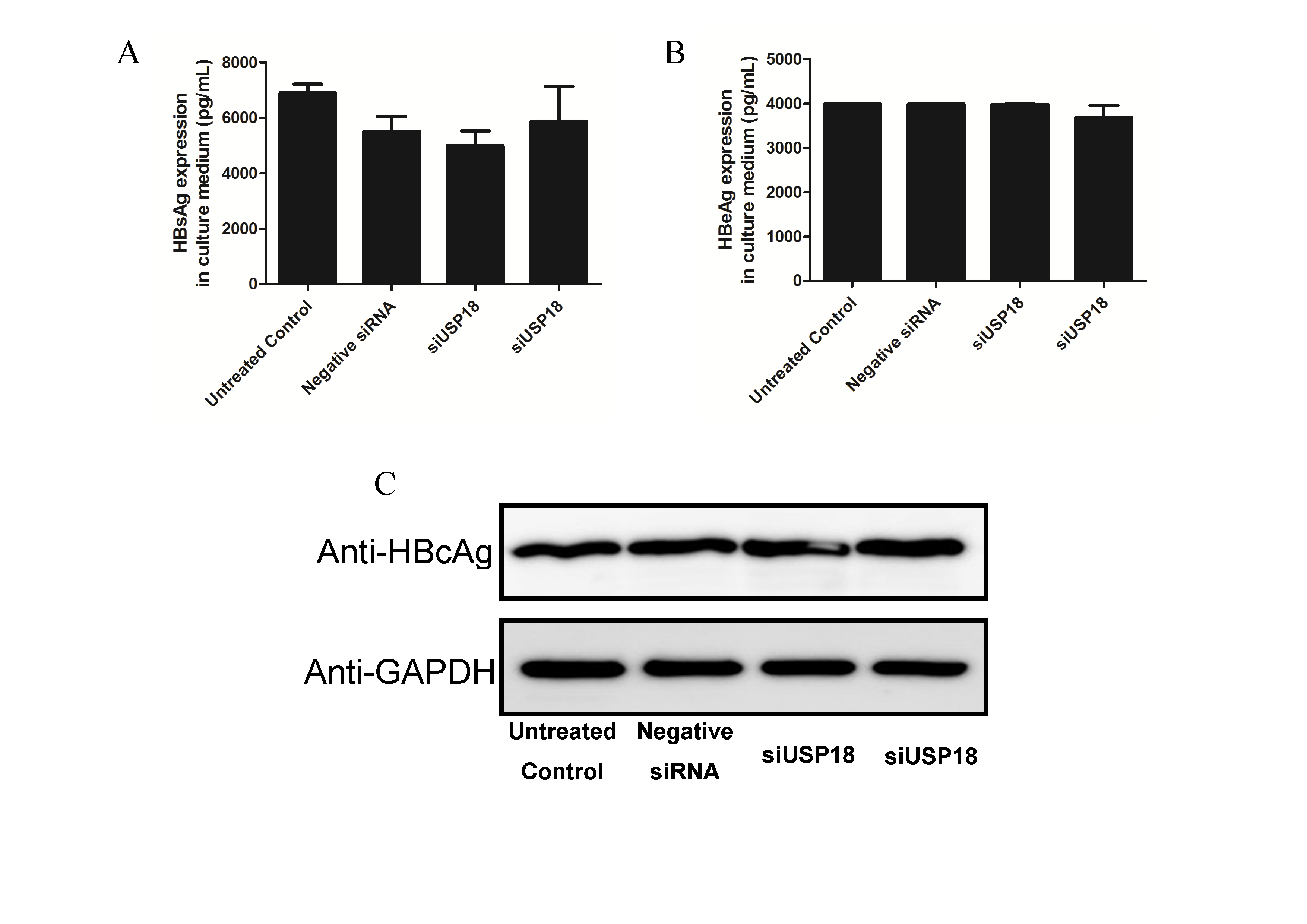
**

**Supplemental Figure 3**

**
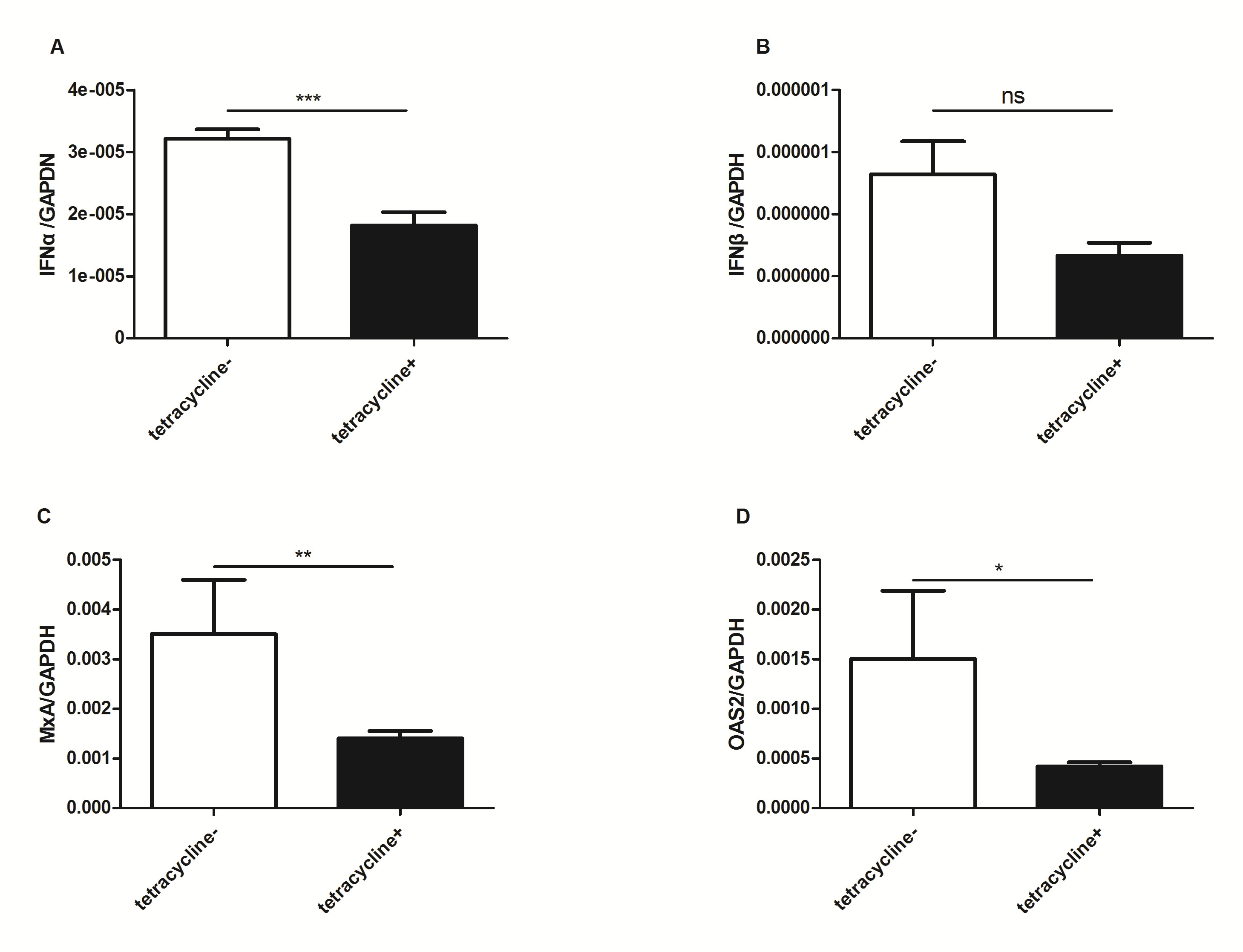
**

**Supplemental Figure 4**

**
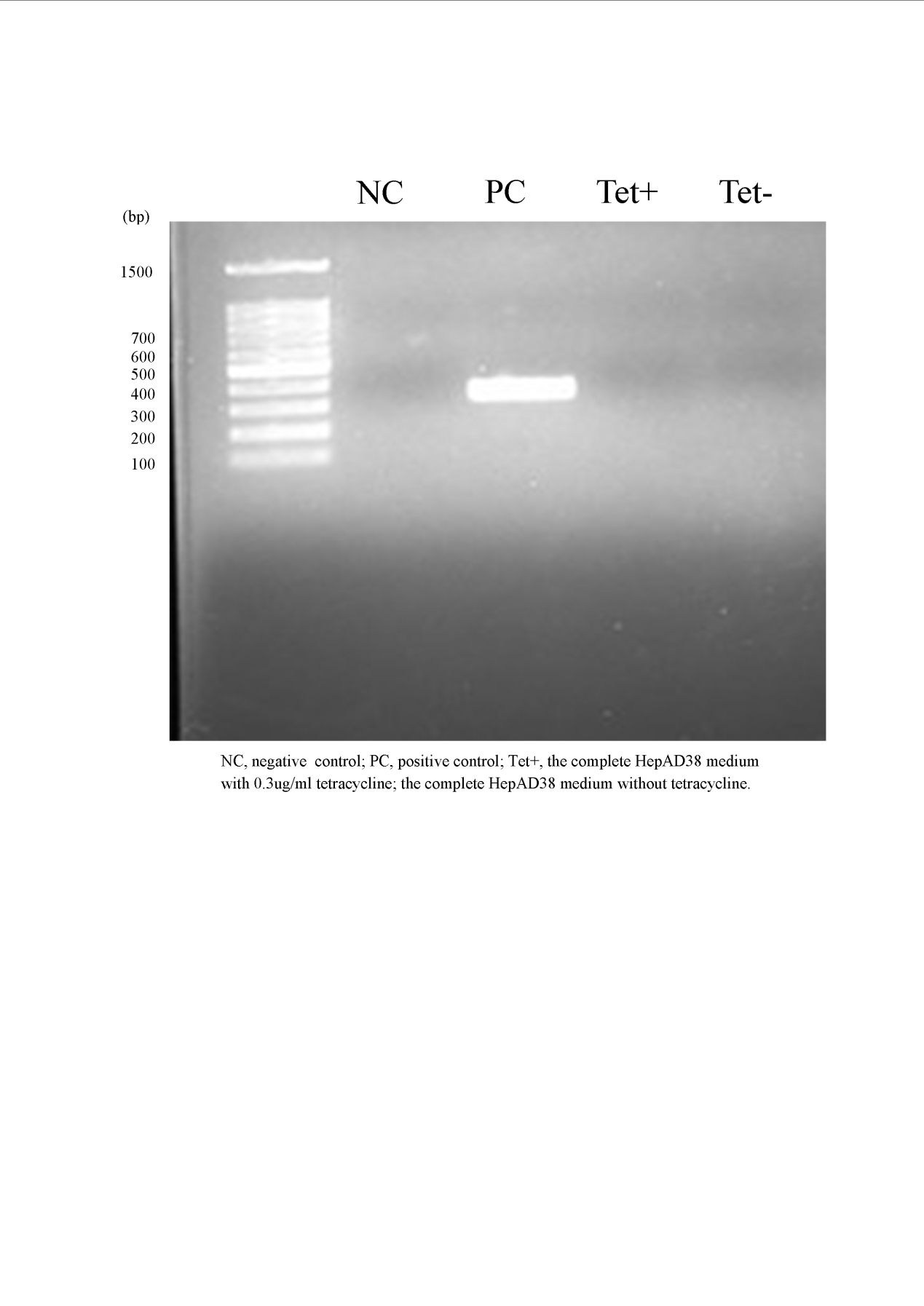
**

**Figure legends:**

**Supplemental Figure 1. Transfection efficiency in HepAD38 cells.** HepAD38 cells were seeded at 3 x 10^5^/ml, 2ml per well in 6-well plates in antibiotic-free medium for 24 hours before 1ug(**A**), 2ug(**B**), 4ug(**C**) GFP plasmid DNA or 4ug empty vector(**D**) was transfected into each well. Fluorescent microscopy images were taken 48 hours post transfection.

**Supplemental Figure 2. USP18 knockdown in HepAD38 cells did not affect expression of HBV proteins.** HepAD38 cells were transfected with the 20nM siUSP18, 20nM negative siRNA or left untreated, respectively. Forty-eight hours later, culture medium was collected to quantify HBsAg**(A)** and HBeAg**(B)** expression level by ELISA assay. Intracellular HBcAg was detected by western blot**(C)**. Results are presented as means ± SD(n≥3).

**Supplemental Figure 3. IFN and ISG expression in HepAD38.** HepAD38 cells were grown in the medium with or without tetracycline (1mg/ml) until confluent for 6 days. Intracellular RNA was then extracted. Expression of IFNα(**A**), IFNβ(**B**) and ISGs mRNA including MxA(**C**) and OAS2(**D**) were detected by real-time PCR. Results are presented as means ± SD(n≥3). *p < 0.05; **p < 0.01; ***p<0.001.

**Supplemental Figure 4.** **Detection of mycoplasma in HepAD38 cells.** HepAD38 cells were grown in the medium with or without tetracycline (1mg/ml) until confluent. The supernatant was collected for detecting mycoplasma. NC, negative control; PC, positive control; Tet+, the complete HepAD38 medium with (1mg/ml) tetracycline; the complete HepAD38 medium without tetracycline.
